# Surface damage reduction effect of ultra-high working face in Shangwan coal mine

**DOI:** 10.1038/s41598-024-72261-x

**Published:** 2024-09-11

**Authors:** L. I. U. Xinjie, Yang Yingming, Zhao Yongqiang, WANG Xiaolong, Liu Gang, Xu Zhuhe

**Affiliations:** 1 State Key Laboratory of Water Resource Protection and Utilization in Coal Mining, National Institute of Clean-and -Low-Carbon Energy, Beijing, 102209 People’s Republic of China; 2 CHN Energy Shendong Coal Group Co., Ltd., Ordos, 017004 People’s Republic of China

**Keywords:** Ultra-high working face, Advancing degree, Ground surface movement, Coal, Fossil fuels, Environmental impact

## Abstract

Green mining is the basic background of high-quality mining, and source reduction is the most important part, among which the optimization of working face height and length is the most active. How to control the working face parameters, reasonably evaluate the surface subsidence characteristics of the super-long working face, and identify the limit working face length and the surface discontinuous deformation threshold is particularly important. In order to solve the problem of irreversible damage to the surface caused by the mining process of the working face, this paper discusses the whole process of surface movement in the 8.8 m super-large mining height working face of Shangwan Coal Mine through field investigation, the maximum subsidence is 6.20 m, with a subsidence coefficient of 0.72. Combined with the inflection point trajectory, advancing degree and subsidence coefficient, the 0.9 coefficient of the working face subsidence basin boundary and the loss reduction strategy far from the limit working face threshold are proposed. The results show that the subsidence basin of 12,401 working face accounts for 34%, the continuous deformation area of surface accounts for 64%, and the influence area of discontinuous deformation area is within 10 times of mining height. With the help of borehole detection verification, the caving zone height within the coal seam overburden to be between 33.2 and 33.25 m and the fracture zone height between 118.08 and 132.83 m. Therefore, the caving ratio is about 3.8, and the fracture ratio is about 13–15, which provides a strong basis for the optimization design of working face and the timing of surface ecological management.

## Introduction

Coal stands as the cornerstone of China's energy strategy, playing a pivotal role in the nation's rapid economic growth and ensuring energy security. In 2023, China's national coal output soared to 4.71 billion tons. Notably, the combined production from Shanxi, Shaanxi, and Inner Mongolia amounted to 3.83 billion tons, constituting an impressive 81.3% of the total production. This statistic not only underscores the pivotal role of these regions in China's coal industry but also highlights their efficiency and productivity. With an average production capacity of 2.51 million tons per year for each mine in these areas, they far surpass the national average of 1 million tons per year.

The interplay between mining operations and overburden integrity is critical for maintaining environmental and structural safety. Recent studies have sought to quantify this relationship by examining the impact of various mining parameters on overburden damage. He et al.^1^ established a quantitative relationship by analyzing the effects of surface length and advancing speed on the extent of overburden damage, as well as the development of water-conducting fracture zones. This foundational work provides a basis for predicting and mitigating damage due to mining activities.

Further expanding on this, Li et al.^2,3^ developed a damage dissipation model that delineates the relationship between the damage coefficient and mining space across the different zones of overburden, categorized into 'three zones.' Their research underscores that source reduction is paramount in controlling mining-induced damage. Specifically, they found that crack widths exceeding 20 cm predominantly exhibit a vertical negative effect, significantly increasing soil erodibility within a horizontal span of 83 cm, a finding supported by Wang et al.^4^.

Technological advancements have also played a pivotal role in monitoring and analyzing mining impacts. Zhang et al.^5^ utilized an integrated approach employing InSAR, GNSS, and 3D laser scanning technologies to monitor surface subsidence at the Shendong Shangwan Mine. Their work effectively maps the distribution characteristics of surface subsidence, offering invaluable insights for managing and mitigating mining-related surface damage.

Hu et al.^6^ offered a comprehensive ground stability assessment framework integrating field, D-InSAR, geophysical, and geological methods, precisely mapping subsidence zones and validating its rationale. However, its applicability is area-specific, necessitating adaptations for broader contexts. Wojciech et al.^7^ improved surface deformation forecasting in underground mining by estimating Knothe's model parameters and quantifying forecast uncertainties, enabling safety-centric margin reductions through Monte Carlo or uncertainty propagation. Their Polish coal mining case study demonstrated the approach's effectiveness. Sakhno et al.^8^ underscored the exacerbated ground movement hazards from longwall goaf flooding in the Ukrainian Donbas, emphasizing the urgency of timely prediction and surface control measures. Lastly, Chi et al.^9^ enhanced mining subsidence prediction accuracy in thick unconsolidated layers by introducing an exponential influence coefficient and unconsolidated layer factor, validated in the Huainan mining area, aligning predictions with observations.

As China's coal development strategy shifts westward, the Yellow River Basin emerges as a critical area where the high demands of coal mining clash with the need for environmental preservation. This region showcases a striking contradiction: the pursuit of intensive mining operations against the backdrop of a fragile ecological landscape. Research, such as that by Zhang et al.^10^, suggests that despite the adverse effects of mining, the basin's damaged ecosystems exhibit a "self-healing" capability, powered by natural processes.

In the Shendong mining area, this dichotomy between mining-induced damage and ecological recovery is particularly evident. Despite extracting a staggering 3.5 billion tons of coal and establishing China’s first 200-million-ton coal production base, remarkable efforts in ecological management have led to substantial environmental restoration across 473 square kilometers of mining land. From an initial vegetation coverage of merely 3–11%, proactive ecological interventions have skyrocketed greenery levels to 64%. This transformation has not only revitalized the transition zone between the Loess Plateau's hilly and gully regions and the Mu Us Sandy Land but also fostered an oasis where there was once desert and gobi. Today, what used to be barren landscapes are now vibrant ecosystems, with "rocky mountains" turning into "green water and green mountains."

The concept of green mining emphasizes a paradigm shift towards reducing the environmental footprint of coal extraction. By leveraging generalized resources, this approach seeks to minimize ecological disturbances at their origin through strategic control over the strata's breaking movements during mining and the efficient utilization of coal seams and associated resources (Xu,^11^^,^Xu,^12^. This paper aims to quantify the capacity for mining loss reduction by examining the interplay between mining operations and surface responses in the Shendong mining area, specifically focusing on operations that annual coal production of 200 million tons.

Our research centers on the 12,401 super-large mining height (8.8 m) working face of the Shangwan Coal Mine, selected for its representativeness in the context of large-scale coal extraction. By analyzing surface subsidence patterns and morphological characteristics associated with this working face, we have been able to scientifically determine the optimal 'amount' (length of the working face) and strategies for green reduction (mitigating non-uniform settlement). These findings hold substantial importance for achieving source reduction and facilitating surface ecological improvement in the context of intensive mining practices prevalent in western China’s mining regions.

## Mining surface monitoring of Shangwan 12,401 working face

The process of underground engineering inevitably leads to significant changes in the stress distribution and displacement of the overlying strata, presenting a complex interplay of forces. Filling mining in goaf is the most environmentally friendly mining method. However, the annual output of a working face in Shangwan Coal Mine is 16 million tons, while the current annual filling capacity is only 2 million tons. It is difficult to balance filling and efficient mining. In fact, the surface of the 12,401 working face is sandy soil, and there are no other resources and buildings on the surface. The surface of high-intensity mining has shown self-repairing ability, such as the self-healing of some cracks, and the growth of vegetation has not been greatly affected.

This study aims to explore the surface damage reduction effect facilitated by the implementation of a super-long working face with a large mining height, specifically within the Shangwan Coal Mine. By comprehensively understanding the entire trajectory of surface movements associated with mining activities, this research seeks to elucidate the dynamic response relationship between the mining operations and the surface reactions.

**Fig. 1:**
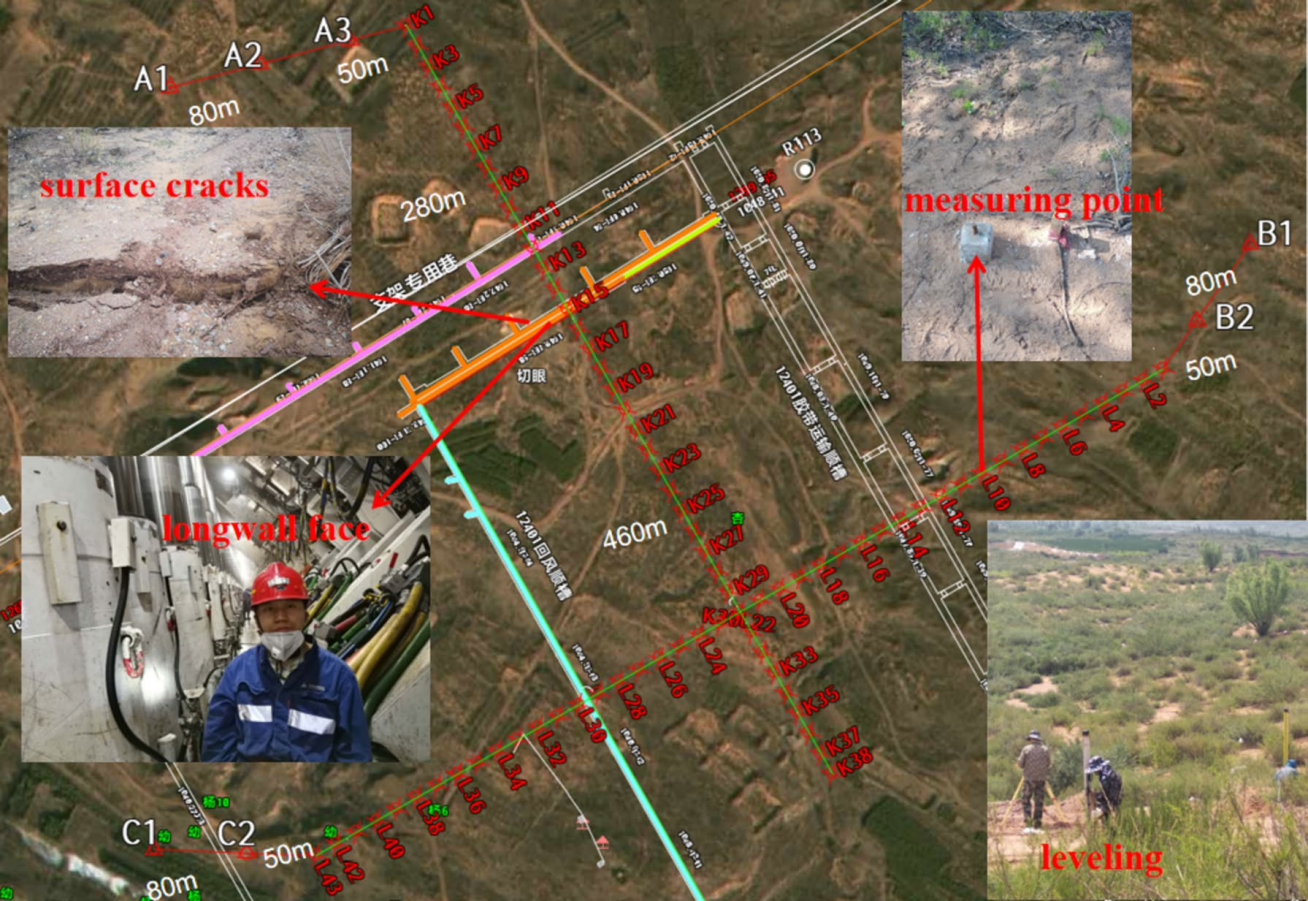
12,401 working face surface movement monitoring design. The background image is sourced from the Ovital Map, where the frame information is derived from the superposition of latitude and longitude coordinate CAD information. The working face is a photo of myself taken at the scene, and the other three photos are taken on site during the field survey.

To achieve this objective, a detailed observation of rock movements was conducted at the 12,401 working face of the Shangwan Coal Mine. The methodology employed involved a strategic arrangement of cross lines for monitoring, as detailed in Fig. 1. This figure illustrates the layout of the observational setup, providing a foundational reference for understanding the spatial dynamics of rock movement in relation to mining activities.

**Fig. 2:**
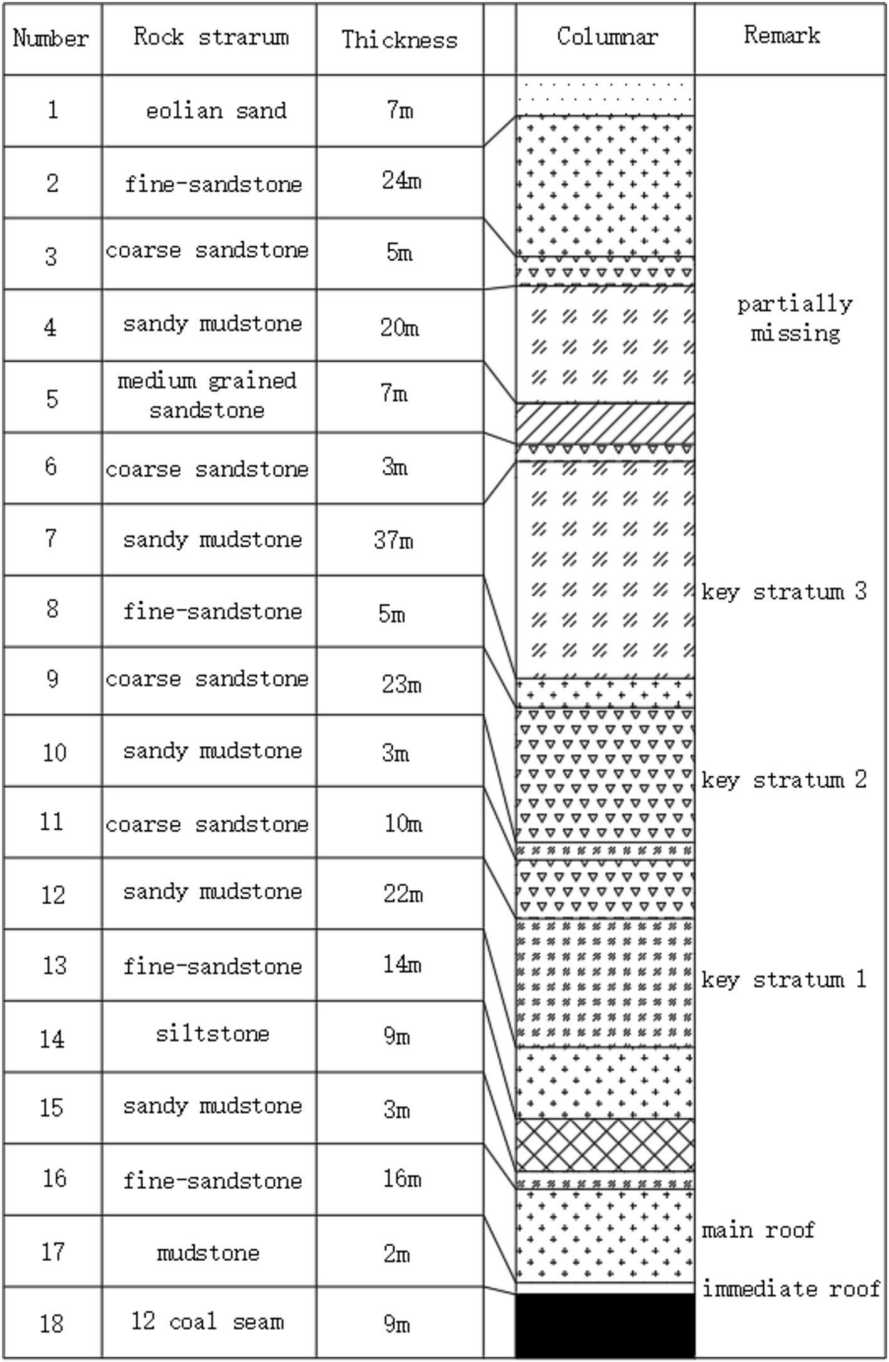
Comprehensive geological of 12,401 working face.

The 12,401 mining operation features a working face with a mining height of 8.6 m, an advancing length of 5286 m, an inclination length of 299.2 m, a dip angle ranging from 1 to 5 degrees, and a buried depth of 210 m, the comprehensive geological as shown in Fig. 2. The layout on the surface adopts a cross-line configuration, with 38 measuring points (K1-K38) positioned along the advancing direction, each spaced 20 m apart, and the inclination line extending 760 m. In the direction of the working face's tendency, 43 measuring points (L1-L43) are arranged, also 20 m apart, along an 860-m tendency line. The intersection of L22 and K30 marks a critical cross measuring point. Permanent control points A1, A2, and A3 are established along the K line, while B1, B2, C1, and C2 are placed along the L line, with control points 80 m apart and 50 m from the measuring points. The installation of the fully mechanized mining face commenced on March 2, 2018, and mining operations began on March 20, 2018. Initial surface movement observations started on February 26, 2018. Over a span of 19 months until August 2019, the project conducted 24 surface movement process monitoring sessions.

## Analysis of advancing direction (strike) measuring points

**Fig. 3:**
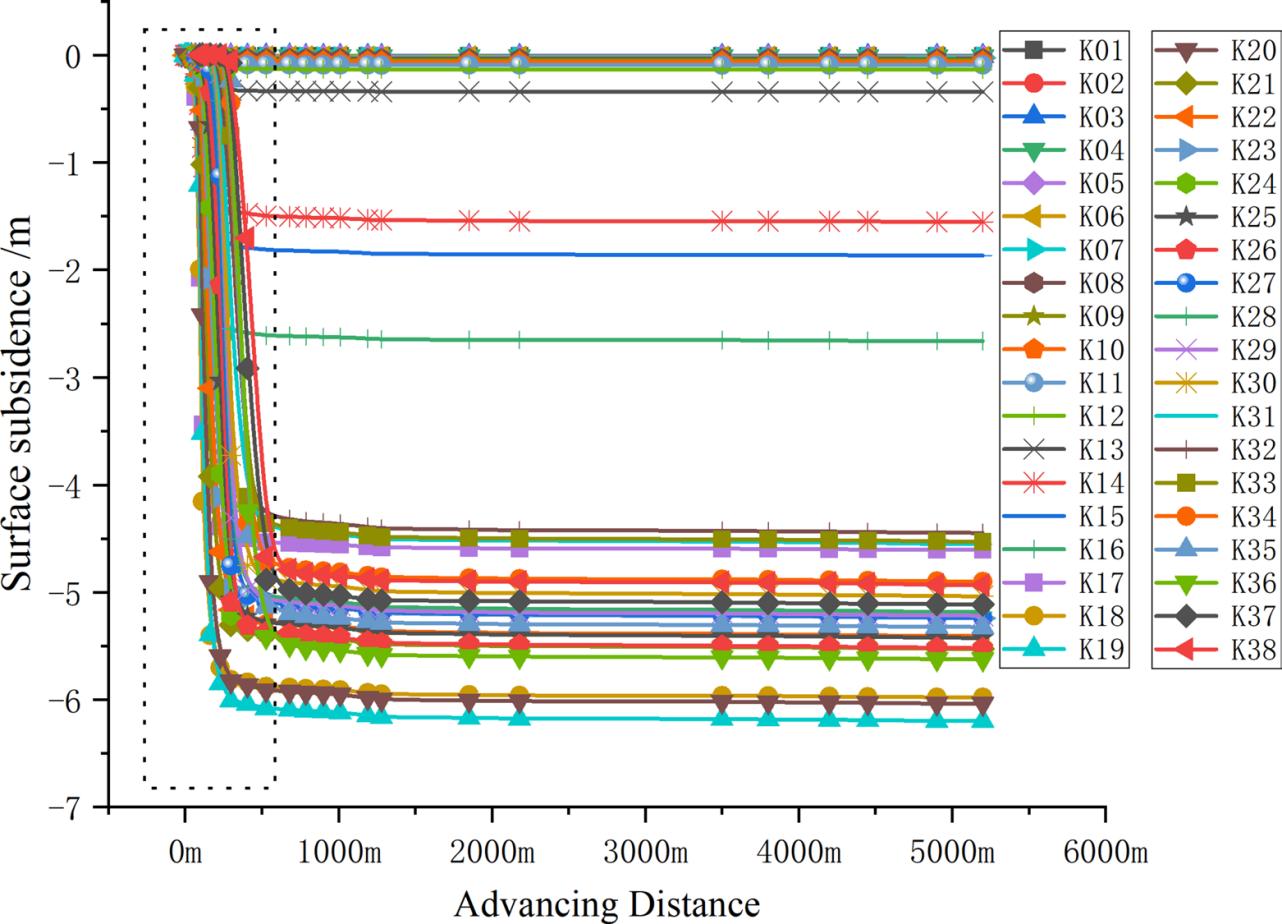
Sinking analysis of 12,401 working face.

To accurately depict the correlation between mining activities and surface subsidence, this analysis utilizes the advancing distance of the working face as the temporal axis. It examines the comprehensive changes across 38 surface monitoring points, ranging from K1 to K38. The resultant surface subsidence curve is presented in Fig. 3.

Subsidence ceases to increase with changes in mining dimensions, exhibiting varying values across different areas of the mining surface. The K19 curve, located within the goaf 80 m from the open-off cut, displays the maximum subsidence of 6.20 m with a subsidence coefficient of 0.72. In contrast, the K15 curve, positioned directly above the cutting eye, registers a subsidence of 1.85 m, approximately 30% of the maximum value, and less than half of the peak subsidence. Surface subsidence beyond the open-off cut diminishes as distance increases, with the most significant drop-off at 80 m (K11) outside the open-off cut, where subsidence reaches a mere 0.09 m, indicating negligible mining impact.

To illustrate the relationship between the advancement degree, a ratio of the advance distance to the buried depth (210 m) and the face length (299.2 m) is utilized, resulting in 400 m/210 m≈1.9 and 400 m/299.2 m≈1.34. This calculation suggests that the 12,401 working face attains full mining status when the advancement degree (ratio) falls between 1.34 and 1.9.

### Mining subsidence and residual subsidence

**Fig. 4:**
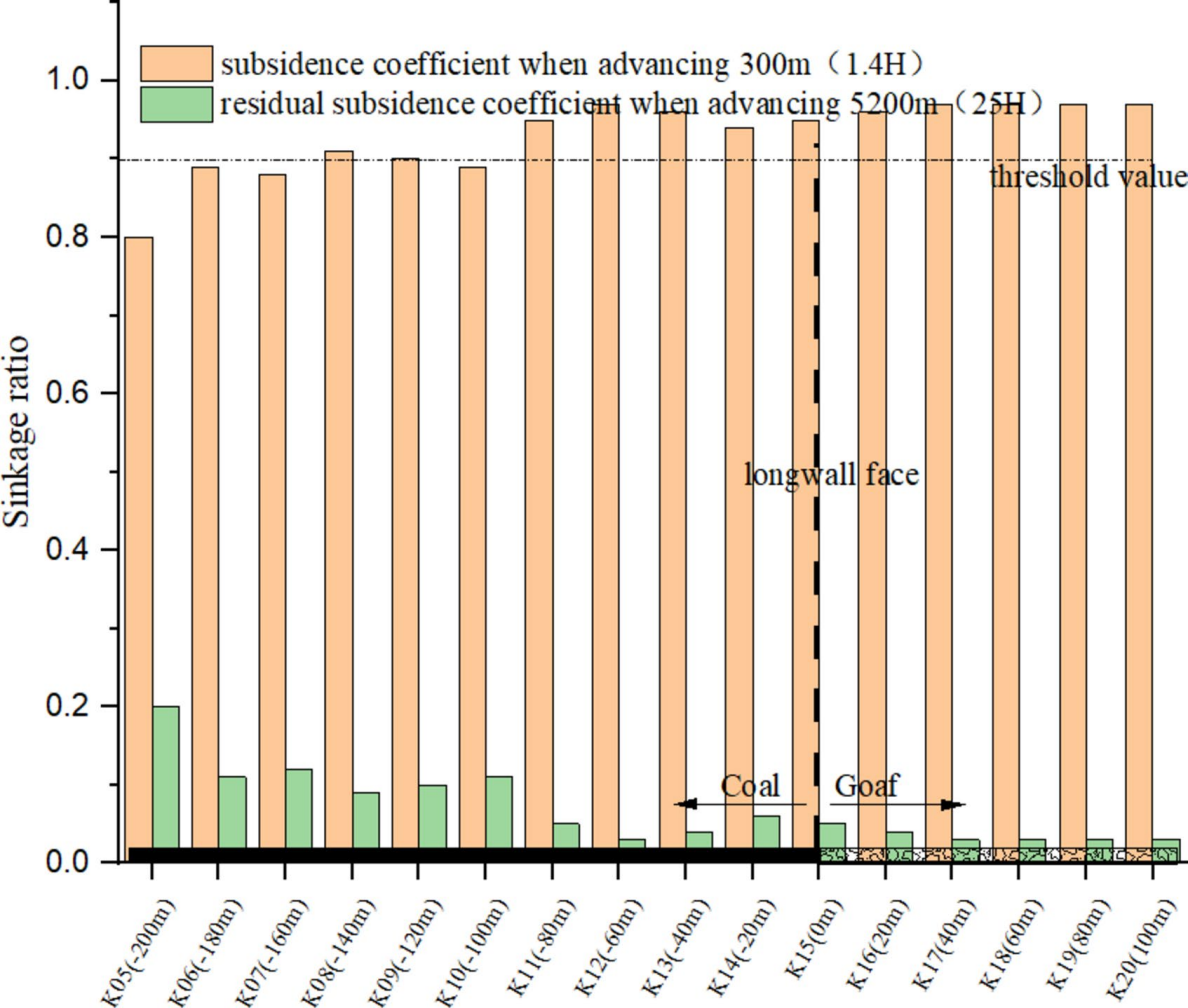
Analysis of the sinking ratio of the advancing distance 300 m and the 5200 m.

The stabilization of surface subsidence, particularly residual subsidence, typically culminates in a final stable state N years post-mining, where N equals the buried depth divided by 100 m. To elucidate the comparative dynamics between mining subsidence and residual subsidence, subsidence data at two critical junctures were analyzed: during a 300-m advancement on May 30, 2018 (with an advancement degree of 1.4), and following the cessation of mining activities on August 12, 2019 (with an advancement degree of 25). This analysis, focusing on a 200-m range around the cutting area, visually demonstrates the ratio of surface subsidence at these times, as depicted in Fig. 4.

When the 12,401 working face advances 300 m, surface subsidence within the − 80 m to 100 m range near the open-off cut comprises over 95% of the final subsidence value. As the distance from the open-off cut increases, the proportion of mining subsidence decreases, yet remains above 80%. This suggests that near the open-off cut, the surface has essentially achieved a state of complete mining subsidence, accounting for 80% to 95% of the total. Consequently, a maximum subsidence threshold boundary can be established at 0.9, indicating that subsidence reaching 90% of the maximum value signifies full mining or complete subsidence.

The mining boundary surface typically exhibits an S-shaped subsidence curve, characterized by discontinuous deformation zones and uniform subsidence zones, significantly impacting surface vegetation (Liu et al.,^13^. The inflection point of the curve marks the transition between these zones. To delineate the boundary between discontinuous and continuous subsidence, the second derivative of the S-shaped curve is set to zero, with the inflection point defining the discontinuous deformation boundary.

### Trajectory analysis of advancing direction inflection point

**Fig. 5:**
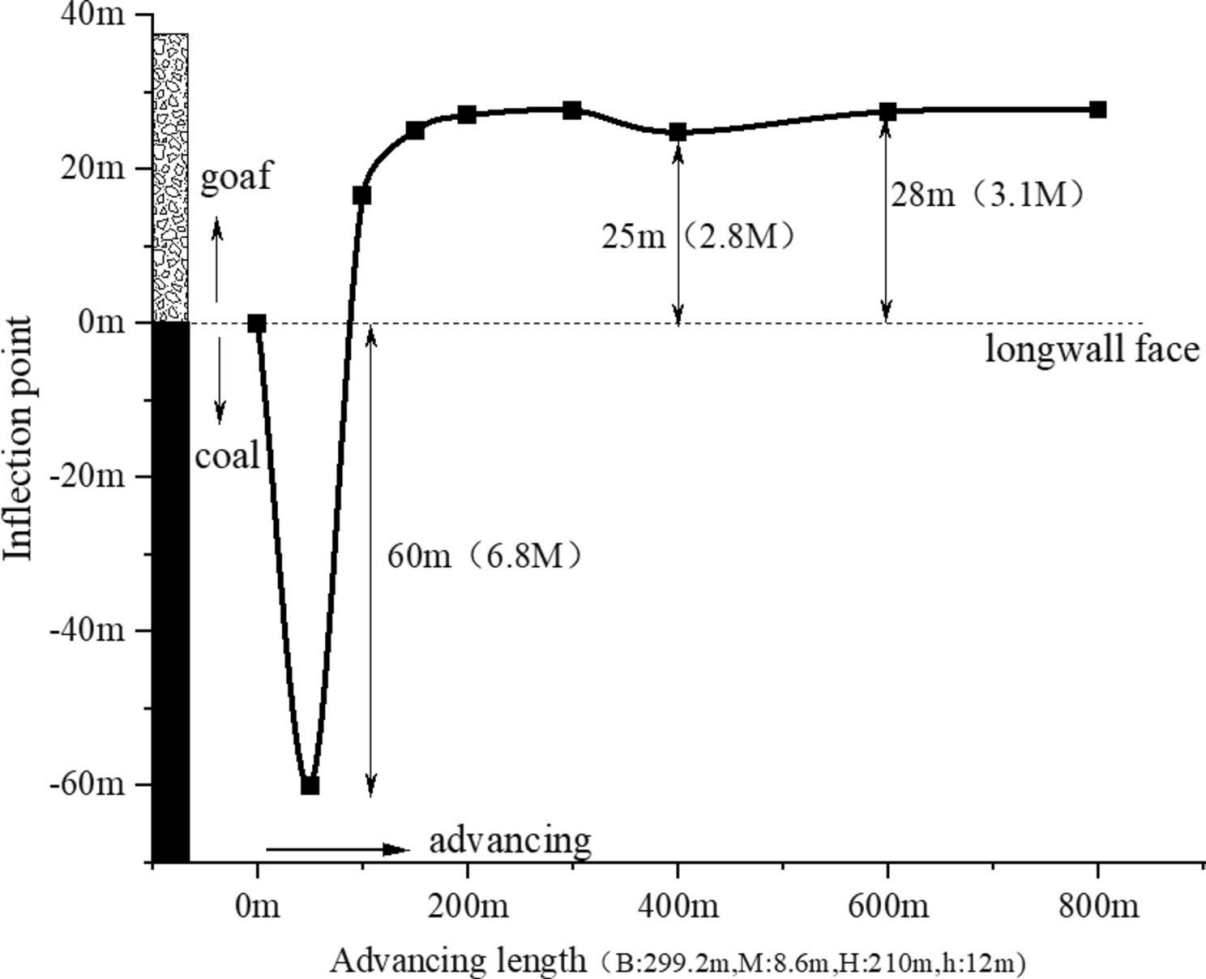
Inflection point trajectory of trend subsidence curve.

Surface subsidence achieves a state of full mining when the advance distance reaches 300 m. To comprehensively depict the evolution throughout the entire process, this study selects the dynamic changes in the curve shape characteristic of the interval from the working face's open-off cut to an advance of 800 m (advance degree of 3.8). The trajectory of the inflection point is illustrated in Fig. 5.

In Fig. 5, B represents the length of the working face, M the mining height, H the buried depth, and h the thickness of the loose layer. Initially, when the open-off cut is 10 m, equivalent to an advance distance of 10 m, the inflection point's position is approximately zero, suggesting negligible surface impact. As mining progresses, surface changes become apparent, with the inflection point swiftly moving beyond the mining boundary. At an 80-m advance, the inflection point peaks at 60 m outside the open-off cut, indicating that the subsidence curve within the 0–80 m advance range is nearly a monotonically increasing state without significant shape alteration. Beyond this advance, the inflection point gradually moves from outside to inside the cut, signifying surface discontinuous deformation post an 80-m advance and a subsequent change in the subsidence curve's shape. The inflection point returns to zero at a 100-m advance, eventually stabilizing between 25 and 28 m within the goaf. The trajectory of the inflection point simplifies to a path of 0 m → -60 m → 0 m → 25 m → 28 m.

Thus, the primary influence boundary of the 12,401 working face on the surface extends to 60 m outside and 28 m inside the open-off cut. Due to the presence of three key strata above, the working face exhibits a "two large and one small" pressure step distance pattern, with an average pressure step distance ranging from 7 to 14 m. A periodic variation is completed within the 200 m range, converging with the trajectory changes of surface inflection points.

## Analysis of working face inclination measuring points

### Analysis of limit working face length

**Fig. 6:**
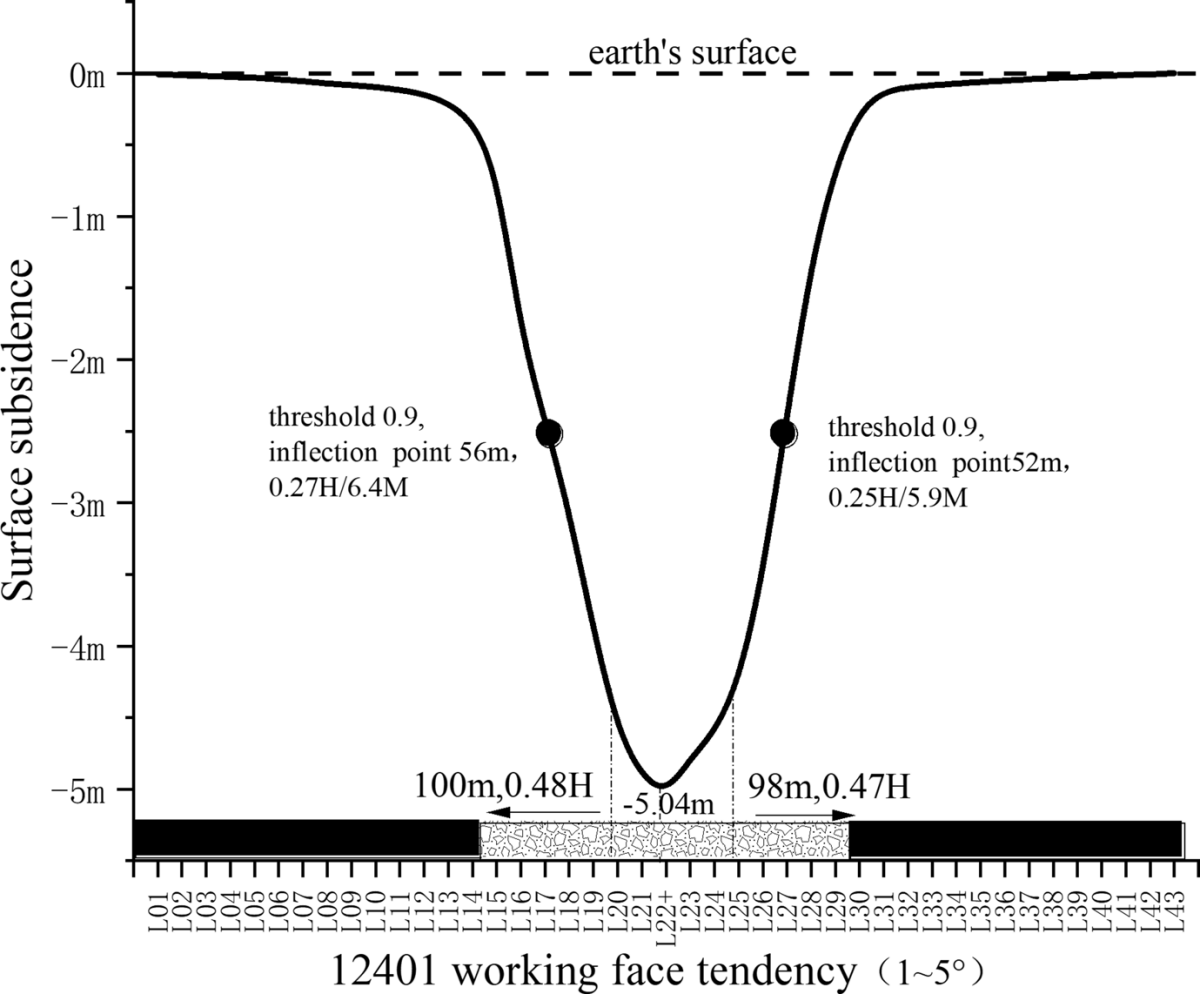
Tendency sinking curve characteristic diagram.

A threshold of 0.9, relative to the maximum subsidence value mentioned previously, is utilized to delineate the boundary of the fully subsided basin. This study analyzes data from 43 measurement points associated with the subsidence trend of the 12,401 working face, as illustrated in Fig. 6.

The maximum surface subsidence recorded along the strike survey line is 5.04 m, with the threshold boundary calculated as 5.04 × 0.9 = 4.5 m. Analysis of the diagram data reveals that the cumulative length contributing to the full subsidence of the working face is 100 m + 98 m = 198 m, indicating that the critical length for the working face is 198 m. Consequently, the effectiveness of reducing losses at the working face increases with its length exceeding the threshold of 198 m. For instance, the 12,401 working face, measuring 299.2 m, results in a fully subsided basin boundary of 299.2 m—100 m—98 m = 101.2 m. Thus, the basin's length represents 34% of the 12,401 working face's total length, calculated as 101.2 / 299.2 = 34%.

Therefore, if the length of the working face is greater than 198 m, the longer the working face, the more the proportion of the basin width, and the better the effect of the average settlement reduction. If the length of the working face is less than 198 m, the shorter the working face, the better the damage reduction effect.

### Trajectory analysis of the working face inclination inflection point

The dip angle of the 12,401 working face ranges from 1 to 5 degrees, and the locations of the inflection points within the two crossheadings are slightly different, ranging from 52 to 56 m into the goaf. This indicates that the range of discontinuous deformation within the crossheading does not exceed 56 m. Consequently, the length of the area experiencing continuous deformation on the working face is calculated as 299.2 m − 56 m − 52 m = 191.2 m, with this continuous deformation zone comprising 64% of the total length of the working face, as determined by the ratio 191.2/299.2 = 64%.

**Fig. 7:**
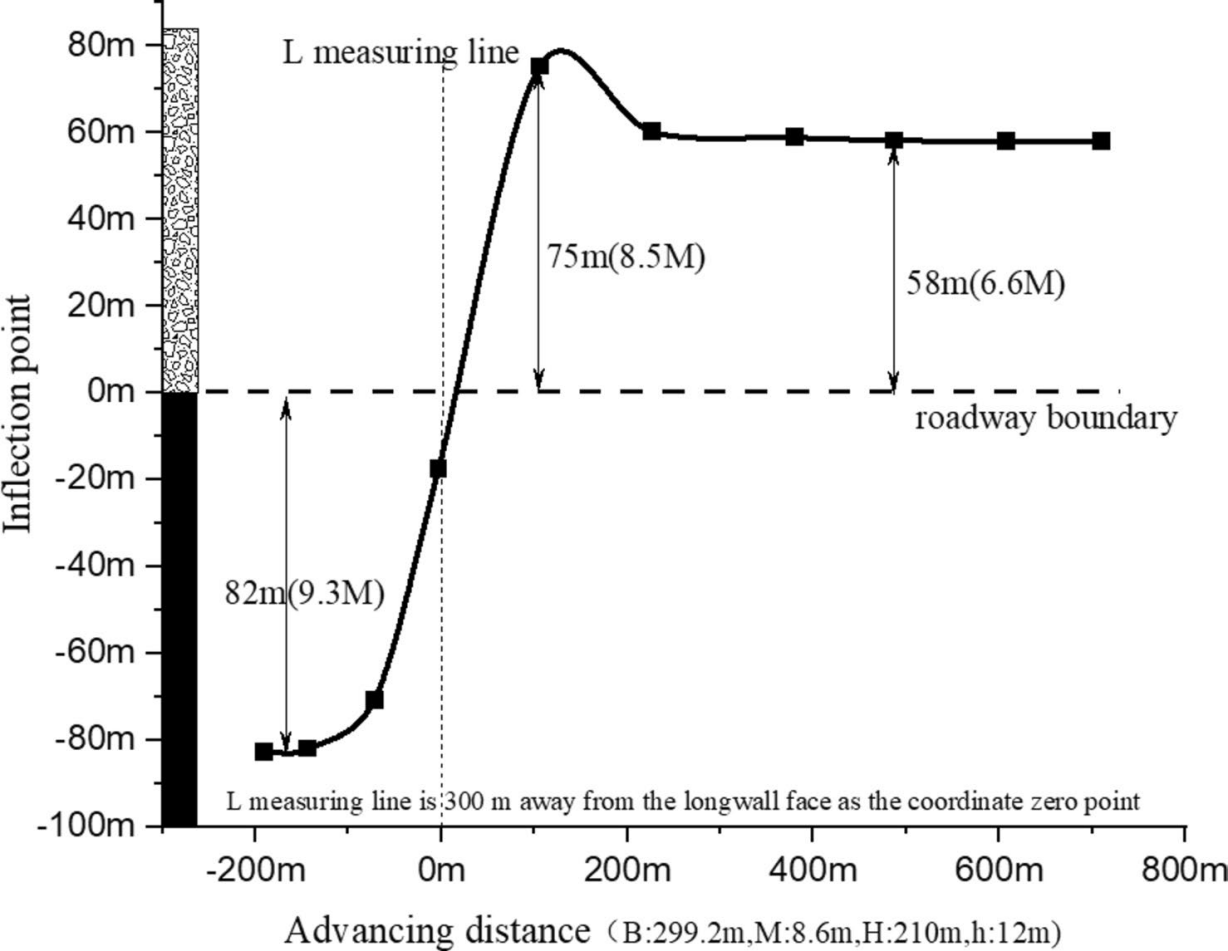
Tendency sinking inflection point trajectory diagram.

Furthermore, it is noted that the curve measuring line is situated 300 m from the open-off cut. To elucidate the trajectory changes of the inflection point on the tendency curve, the morphological characteristics of the subsidence curve along the tendency measuring line, across propulsion distances ranging from 100 to 800 m, are compared and analyzed, as depicted in Fig. 7.

Analysis of the trend trajectory reveals a rapid shift in the inflection point of the tendency, initially from zero to beyond the mining perimeter, then swiftly moving towards the goaf side, and ultimately stabilizing at 58 m within the goaf. The progression of the inflection point follows a path from 0 m to − 82 m, back to 0 m, then to 75 m, and finally to 58 m. Consequently, areas 82 m beyond the roadway boundary and 75 m inside the goaf are identified as part of the mining's influence boundary.

## Analysis of overburden rock characteristics

### Borehole design

**Fig. 8:**
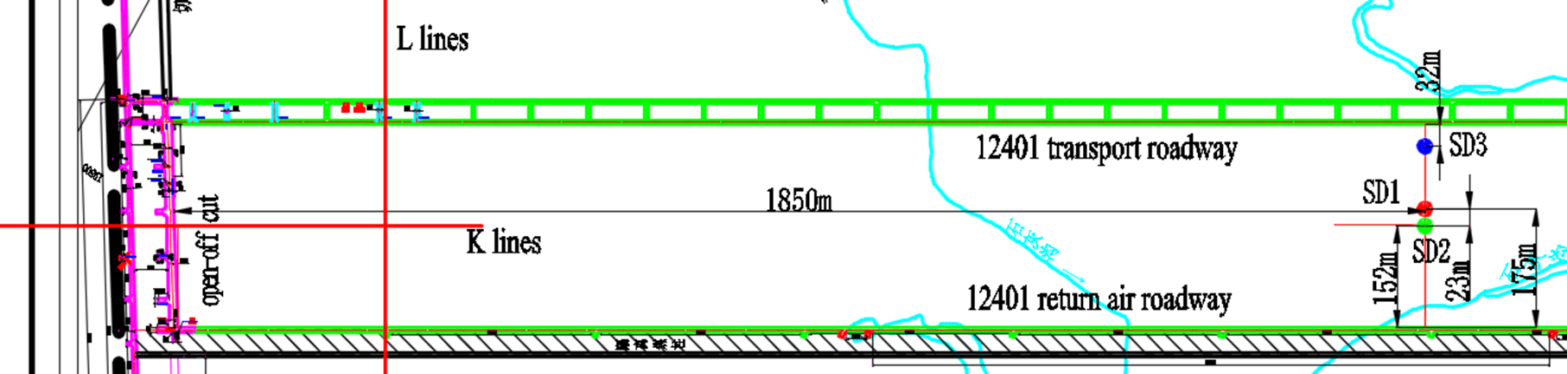
Borehole layout diagram.

To examine the movement characteristics of the overlying strata at the 12,401 working face, as documented by Yang^14,15^, three boreholes were drilled during the mining process. Positioned approximately 1850 m from the cut hole, these boreholes are distributed in an asymmetrical "line" pattern. Borehole SD1, constructed prior to mining, is situated 175 m from the return airway and 125 m from the main haulage roadway. The boreholes SD2 and SD3, drilled after mining commenced, flank the pre-mining borehole. Their precise locations are depicted in Fig. 8 and detailed in Tables 1 and 2.

**Table 1:** List of basic situation of drilling holes.

Number	Termination depth	Quality	logging depth	Beginning date	Completion date	Loose layer	Bed rock	Fracture zone
SD1	187.43 m	A grade	186.70 m	2018.09.19	2018.09.24	69.6 m	98.5 m	
SD2	177.25 m	B grade	164.70 m	2019.03.5	2019.03.17	44.4 m	83.6 m	46.8 m
SD3	182.52 m	B grade	174.80 m	2019.03.19	2019.03.27	41.4 m	74.3 m	72.0 m

**Table 2:** Observation Data of Pre-mining Borehole SD1 (As of April 30, 2019).

Measuring point	Lithologic	Depth (m)	Distance from the top of coal seam (m)	Relative displacement (m)	Absolute displacement (m)
Point 1	Fine sandstone	41	126	0.074825	4.751825
Point 2	Fine sandstone	57	110	0.125876	4.802876
Point 3	Coarse sandstone	68	99	1.248198	5.925198
Point 4	Fine sandstone	79	88	1.063534	5.740534
Point 5	Medium sandstone	96	71	1.170742	5.847742
Point 6	Medium sandstone	115	52	1.484356	6.161356
Point 7	Coarse sandstone	124	43	1.618336	6.295336
Point 8	Fine sandstone	133	34	1.567089	6.244089
Point 9	Fine sandstone	141	26	1.471666	6.148666

### Drilling data analysis

**Fig. 9:**
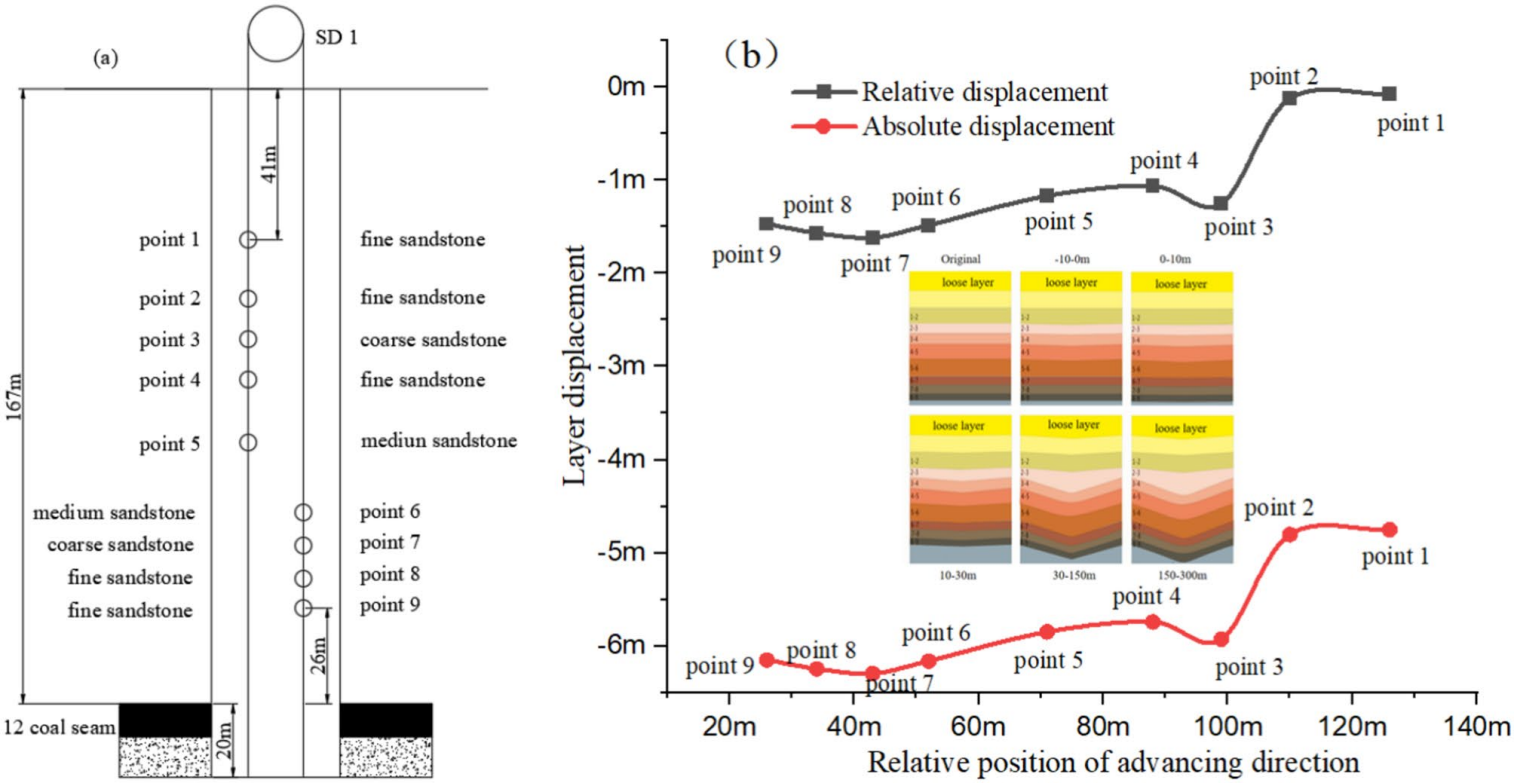
Installation diagram of anchor claw and displacement diagram.

To accurately obtain the stratified subsidence parameters of the overburden rocks before and after the excavation of Working Face 12,401, nine anchor-claw extensometers were installed in pre-mining borehole SD1 (with a total depth of 187 m and a coal seam burial depth of 167 m) to monitor the continuous subsidence of the roof at different depths. The installation depths are 41 m, 57 m, 68 m, 79 m, 96 m, 115 m, 124 m, 133 m, and 141 m, respectively, and the corresponding heights from the coal seam are 126 m, 110 m, 99 m, 88 m, 71 m, 52 m, 43 m, 34 m, and 26 m, respectively. A schematic illustration of the installation sites is presented in Fig. 9a.

Upon the advancement of the working face by 6 m past the borehole—effectively positioning the borehole behind the support top beam—displacements begin to be observed in the overburden of the coal seam. Notably, when the working face extends 10 m beyond the borehole, significant displacement changes are simultaneously detected by the point 5 to point 9 displacement meters (located 26 to 71 m from the roof), indicating potential fracturing or dislocation within the rock mass at the borehole's site. Within the 40 to 100 m range inside the goaf, the point 3 to point 9 measuring points exhibit rapid and synchronized settlement, followed by gradual stabilization, whereas the point 1 and point 2 measuring points, closer to the surface at 110 to 126 m from the roof, show only minimal subsidence.

After the observation hole progresses 180 m into the rear of the goaf, the subsidence of the overburden above the coal seam gradually stabilizes. As recorded up to April 30, 2019, the relative and total surface subsidence at each measuring point is detailed in Fig. 9b.

Analysis of the displacement changes across the measuring points reveals a pattern consistent with the general law of stratum subsidence in the goaf: the displacement is greater in the lower measuring points than in the upper ones, reflecting the stratification of rock layers. Specifically, the smallest overall displacements are observed at the point 1 and point 2, medium displacements at point 3, point 4, and point 5, and the largest subsidence at point 6, point 7, point 8, and point 9 points. Following mining, all overburden strata above the 12,401 face experience a rapid increase in subsidence within the 0–10 m range. Beyond this, from 33 to 69 m after mining, the subsidence rate markedly accelerates for all points except point 1 and point 2, with the most significant rates at point 9 and point 7 points. This pattern highlights two periods of rapid subsidence transition to stability across the overlying strata, spanning 0 to 300 m post-mining. This analysis estimates the caving zone height within the coal seam overburden to be between 33.2 and 33.25 m and the fracture zone height between 118.08 and 132.83 m. Therefore, the caving ratio is about 3.8, and the fracture ratio is about 13–15.

## Conclusions

For the 8.8 m ultra-large mining height working face, the observed maximum subsidence is 6.2 m, yielding a subsidence coefficient of 0.72. Mining-induced subsidence comprises between 80 and 95 percent of the total subsidence observed. A threshold value of 0.9 is proposed to delineate the boundary of the subsidence basin and define the limits of the working face.

The boundary surface of the mining area is depicted by an S-shaped subsidence curve, with the trajectory of the inflection point—where the second derivative equals zero—serving to define the curve's shape. Initially, the inflection point is located outside the mining area, swiftly moving to the goaf's edge before stabilizing within a 28 m range (Approximately 3.2 times the mining height) of the goaf. This position of the inflection point serves as the demarcation for the area of discontinuous deformation.

In the case of the western high-strength working face, there exists a significant potential for damage reduction through the strategic optimization of the working face length. Such optimization has proven effective in mitigating damage. Specifically, for the 12,401 working face, the subsidence basin constitutes 34 percent of the area, while the continuous deformation zone on the surface comprises 64 percent. This highlights the importance of length optimization in reducing subsidence impacts and optimizing mining operations for enhanced safety and sustainability.

## Data Availability

Data supporting the results of this study are available from the corresponding author [liu Xinjie] upon reasonable request.
